# Fruit Battery with Charging Concept for Oil Palm Maturity Sensor

**DOI:** 10.3390/s20010226

**Published:** 2019-12-31

**Authors:** Norhisam Misron, Nisa Syakirah Kamal Azhar, Mohd Nizar Hamidon, Ishak Aris, Kunihisa Tashiro, Hirokazu Nagata

**Affiliations:** 1 Faculty of Engineering, Universiti Putra Malaysia, Serdang, Selangor 43400, Malaysia; mnh@upm.edu.my (M.N.H.); ishak_ar@upm.edu.my (I.A.); 2 Institute of Advance Technology (ITMA), Universiti Putra Malaysia, Serdang, Selangor 43400, Malaysia; 3 Faculty of Engineering, Shinshu University, Wakasato 4-17-1, Nagano 380-8553, Japan; tashiro@shinshu-u.ac.jp; 4 Centre for Global Education & Collaboration, Shinshu University, Wakasato 4-17-1, Nagano 380-8553, Japan; hnagata@shinshu-u.ac.jp

**Keywords:** oil palm, moisture content, fruit battery, load voltage, sensor, fruit maturity

## Abstract

There are many factors affecting oil extraction rate (OER) but a large contributor to high national OER is by processing good-quality fresh fruit bunches (FFB) at the mills. The current practice for grading oil palm fruit bunches in mills is using human graders for visual inspection, which can lead to repeated mistakes, inconsistent evaluation results, and many other related losses. This study aims to develop a fruit maturity sensor that can detect oil palm fruit maturity grade and send indication to the user whether to accept or reject the bunches. This study focuses on fruit battery principle and applying the charging concept to the fruit battery in order to generate significant load voltage readings of oil palm fruit battery. The charging process resulted in amplified load voltage readings, which were 4 times more sensitive to changes as compared to normal fruit battery without charging process. From the load voltage readings, the fruits can be characterized into their maturity grade based on moisture content. It was determined that fruits with moisture content less than 44% and average load voltage, *V*_avg_, between 20 to 30 mV are considered ripe fruits.

## 1. Introduction

The global success of palm oil industry has caused Malaysia’s palm oil industry to be one of the main contributors of national growth domestic product (GDP) and led to the creation of employment opportunities [1]. In 2018, Malaysia continues to be the second largest producer and exporter of palm oil globally after producing 27% or 19.52 million tons out of 72.08 million tons of palm oil. However, that same year, Malaysia’s oil palm industry faced many challenges including lower crude palm oil (CPO) production, lower exports, higher palm oil stocks, and lower palm oil prices [2]. In tandem with the fourth industrial revolution or Industry 4.0, the application of mechanization for oil palm industry is a favorable solution to increase the productivity and quality of palm oil production in order to sustain the industry performance.

Through milling process of fresh fruit bunches (FFB), crude palm oil (CPO) is extracted from the fruits, and the percentage of oil extracted is calculated as oil extraction rate (OER). OER is the percentage of weight of oil physically recovered from a known weight of fresh fruit bunches (FFB) processed.

• OER = Oil recovered/FFB processed × 100%

Oil extraction rate (OER) is used as management tool in assessing the performance of palm oil millers and plantations [3]. There are many factors that can affect OER such as FFB quality processed at mills and efficient milling practices. A study has shown that while oil losses in mills at certain processes contributed to low OER, it is not as significant of a loss as compared to low fruit quality sent to the mills, which gives greater impact to the OER performance [4]. Hence, good-quality FFB are desirable to be processed at the mills to obtain high OER.

One of the main issues that mills are currently facing is low FFB quality received from estates. Palm oil estates are sending low-quality FFB to mills, which include unripe, under ripe, or over ripe (rotten) fruits. FGV had suffered RM80 million losses in 2016 due to settlers sending in unripe fruit bunches that could not be processed [5]. In some cases, the unripe and under ripe bunches that has been harvested are stored for a few days in order to allow the fruit to turn the appropriate color of ripe bunches before sending them to mills. However, these fruits have low oil content and the quality of extracted oil does not meet the standard. In these cases, it is not possible for workers at the screening area to detect the stored fruits because they visually appear as ripe fruits with the appropriate outer surface color.

**Figure 1:**
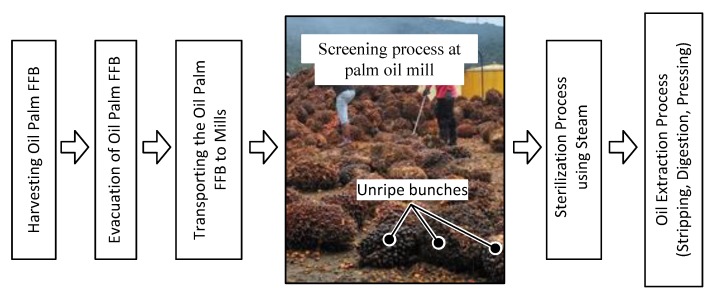
Procedure of oil palm fresh fruit bunches’ (FFB) processing at mills.

It is imperative that good grading and inspection practices are implemented in order to ensure only ripe FFB are sent to the mill for processing. Currently, the grading system in mills is done manually through visual inspection using human graders at the screening process. Figure 1 shows the procedure for processing FFB at mills, which include the manual screening process done by the workers. Through this process, unripe, under ripe, and over ripe FFB are rejected from being processed. However, problems arise when the workers fail to identify unripe and under ripe fruits from the ripe ones due to having similar color. This is a subjective method because human eyes perceive colors differently and it can lead to inconsistent and incorrect evaluation results. Hence, it is not efficient to solely depend on human graders. A systematic and reliable method must be implemented to avoid incurring millions of losses due to sending low-quality FFB to be processed at mills.

For the past few years, several automated fruit grading methods have been proposed and computer vision is one of the most common and popular method [6,7,8]. For this type of system, it requires supporting equipment such as an advanced digital camera and computer software as well as a trained operator (human graders), which are not suitable for efficient on-site testing [9]. The system can also be accompanied by an artificial intelligence system to classify the oil palm FFB [10,11], and researchers have used neural networks and fuzzy regression models for the classification [12,13]. It is known that the method requires a complicated algorithm and precise image collection for the recognition stages.

Spectral analysis is done on the image of the fruit samples and RGB color feature is used to classify fruit maturity, which is based on the different wavelength of the red, green, and blue color of the fruit image [14,15]. These methods depend significantly on the color quality of the image and they can successfully classify the ripe fruits from the bunches correctly based on the average value of red component. However, it is unable to differentiate the red component for unripe and under ripe categories [16]. This method requires human graders to select the samples for the image acquisition procedure and the classification of sample must be performed indoors. Another method that utilizes RGB space is known as the photogrammetric grading system, which requires regular imaging technology components such as image acquisition, image pre-processing, and image segmentation [16,17]. This system is another type of indoor testing where the support equipment for capturing the images can be installed.

Magnetic resonance imaging (MRI) and bulk nuclear magnetic resonance (NMR) are other methods proposed by researchers to monitor the development and ripeness of FFB [18]. Both types of equipment were used to measure the continuous change in the spin–spin relaxation times (T2-values) of the protons in the water and lipids for the development of a ripening tracking process for FFB. However, this method also requires expensive equipment that requires skilled personnel to operate it and is only suitable for indoor laboratory testing.

Another significant method to characterize the maturity grade of oil palm fruits was introduced recently [19], which uses the fruit battery concept. The fruit battery can measure the changes occurring in the oil palm fruit electrolyte properties from decreasing moisture content as the fruit ripens. The ripe fruits flesh displays non-electrolyte properties after being converted to lipids. Thus, lower load voltage values are observed for ripe fruits compared to unripe fruits. The study focused on finding a suitable depth and interval of the electrodes used in fruit battery in order to properly classify the maturity grade. Based on the load voltage readings, a regression analysis was performed, and it was found that the load voltage is strongly related to the moisture content of oil palm fruits.

This study focuses on developing a method that can classify the oil palm fruits maturity grade in order to send indication to the workers whether to accept or reject the fruit bunches at the screening area. The method is convenient and efficient for use in on-site testing right before processing inside mills. In this paper, an electrochemical sensor based on the fruit battery principle is developed to provide indication of oil palm fruit maturity grade. The sensor can be used by manually pricking the fruit bunches during the screening process and load voltage readings are detected by the system. The sensor sends a signal as to whether the fruit is ripe or otherwise and the user can decide to accept or reject the fruit bunches for processing. The signal can easily be understood by the workers conducting the screening process. 

It is important to understand that this method depends on manual visual inspection based on FFB color as a preliminary screening as well as an electrochemical sensor that provides apparent indication of the fruit maturity grade. Determination of fruit maturity grade during the screening process is very similar to the harvesting process method whereby both require visual inspection of the color of FFB. In addition to this method, the determination of the number of loose fruits found on the ground is another method used before harvesting is carried out. Meanwhile, at the screening area, the number of loose fruits sockets found on the bunches is another type of indication for the maturity grade that is suitable to be used for on-site testing. During the screening process, unripe FFB are easily rejected through color inspection due to the apparent black/dark purple color of the fruits, while over ripe FFB show significant number of loose sockets on the bunches. Therefore, determination of these two categories of fruit maturity grade using the proposed sensor is not critical. However, difficulty arises when under ripe and ripe FFB have very similar color and number of loose fruits sockets on the bunches. In addition, it is also possible for estates to send stored FFB that have the appearance of ripe fruits. Hence, application of the sensor is required to screen out these bunches.

In this paper, a new concept of charging of fruit battery is introduced in combination with the basic principle of fruit battery. To simplify, the oil palm fruits used in this study are subject to charging procedure before being used as normal fruit battery. The purpose of this method is to increase the sensitivity of load voltage readings by producing amplified values as a result of charging. This can ensure that the method developed in this research is able to produce significant load voltage characteristics for each oil palm maturity grade. 

In addition, moisture content of each type of fruit is tested to further classify the load voltage readings according to their maturity grade. Determination of moisture content provides quantitative results for the classification, which can replace the visual color inspection method. This method can be performed indoors as a preliminary step to develop the classification. As stated in [19], load voltage is strongly related to the moisture content of oil palm fruits. Despite this, it is not efficient to conduct this testing on-site during screening process at the mills. Using the calculated moisture content for each maturity grade, the method behind the sensor can be developed based on their respective load voltage characteristics.

## 2. Basic Concept of Fruit Battery and Its Charging Concept

### 2.1. Principle of Fruit Battery

**Figure 2:**
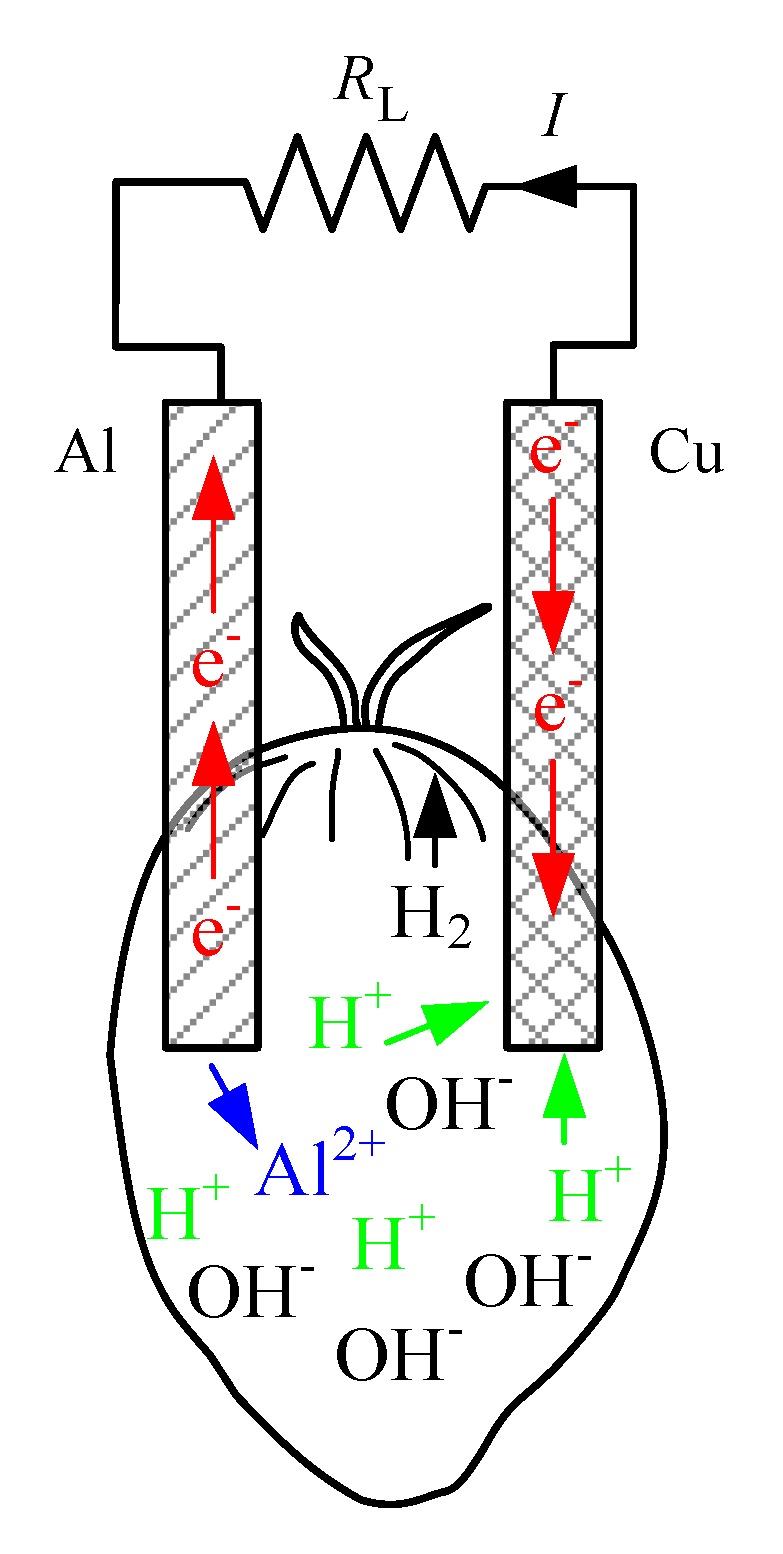
Schematic diagram of oil palm fruit battery electrochemical cell.

A fruit battery is a type of electrochemical cell where two metallic electrodes are thrusted onto the fruit, thus allowing the chemical reaction that takes place to generate electricity. During the chemical process reaction, the more reactive electrode (anode) loses electrons in the oxidation process while the less reactive electrode (cathode) receives electrons in the reduction process. This chemical reaction also applies when oil palm fruit is used in fruit battery experiment. An example of oil palm fruit battery is shown from Figure 2, which illustrates that when aluminum and copper electrodes are used for the experiment, the transfer of electron occurs from the aluminum electrode (anode) to the copper electrode (cathode) through external electric current flow. As the copper electrode receives electrons and combines with the hydrogen ions in the fruit, hydrogen gas is formed and released. Movement of electrons generates electric current, which allows the oil palm fruit to behave as s fruit battery. The chemical reaction can be written as below.

Al → Al^2+^ + 2e^−^ (1)

2H^+^ + 2e^−^ → H_2_ (2)

### 2.2. Basic Concept of Fruit Battery with Charging

The fruit battery can be further completed by using a voltage reader in order to interpret the load voltage readings. However, an oil palm fruit battery alone only produces small load voltage values and they are not sensitive enough to detect changes in voltage readings when parameters of the experiment are varied. For example, the load voltage results obtained in [19] can vary from 10 to 150 mV for all maturity grade when Zinc and Copper electrodes are used in the fruit battery cell. However, when the electrodes are switched to aluminum and copper, as applied in this study, the load voltage readings are only up to 50 mV. Consequently, very similar load voltage values for different types of oil palm fruits cannot be used to provide a good classification of fruit maturity grade. Hence, the theoretical focus of this research is to introduce the concept of charging a fruit battery. When the fruit is charged before the fruit battery circuit is turned on, it behaves similarly to a rechargeable battery. During the charging process, the flow of electron from the negative end (anode) to the positive end (cathode) is reversed and the electron is forced to flow back from cathode to anode. Thus, electrons will bond again with ions at the anode, and the chemical energy of the fruit is restored back. Once the charging process is completed, it is assumed that the fruit is storing high energy, which will be discharged when the fruit battery circuit is turned on.

**Figure 3:**
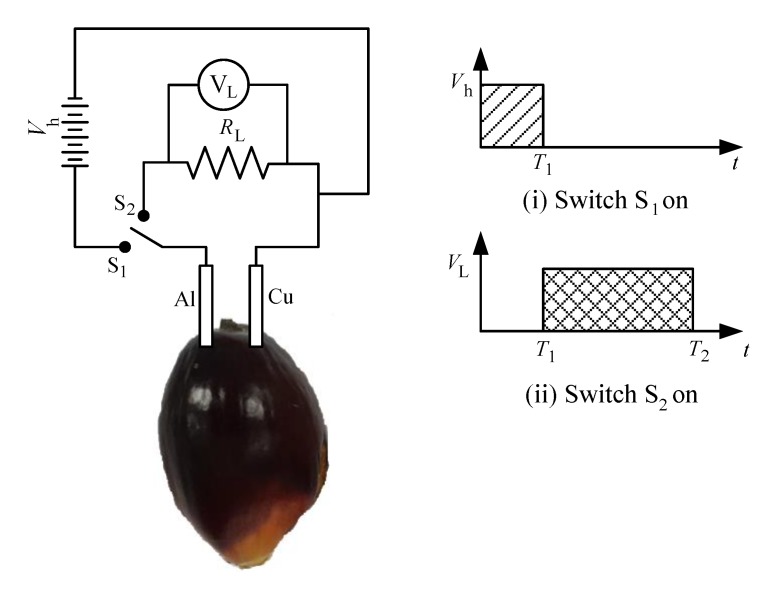
Schematic diagram of fruit battery circuit with charging concept.

From Figure 3, the schematic diagram of the fruit battery circuit with charging concept can be further understood. When the power supply indicated by *V_h_* is turned on and switch *S*_1_ is connected, electrical current of voltage *V_h_* flows through the circuit and charging of fruit occurs as electrical energy is converted to chemical energy inside the fruit. Immediately after the power supply is turned off and switch *S*_2_ is connected for a specific amount of time, the circuit behaves like a normal fruit battery where the chemical energy is converted to electrical energy and the amplified load resistance voltage readings, *V_L_* are generated. These readings illustrate the characteristics of energy discharge from the fruit and it shows an amplified value at the moment of release due to the high energy stored in the fruit as received from the charging process.

**Figure 4:**
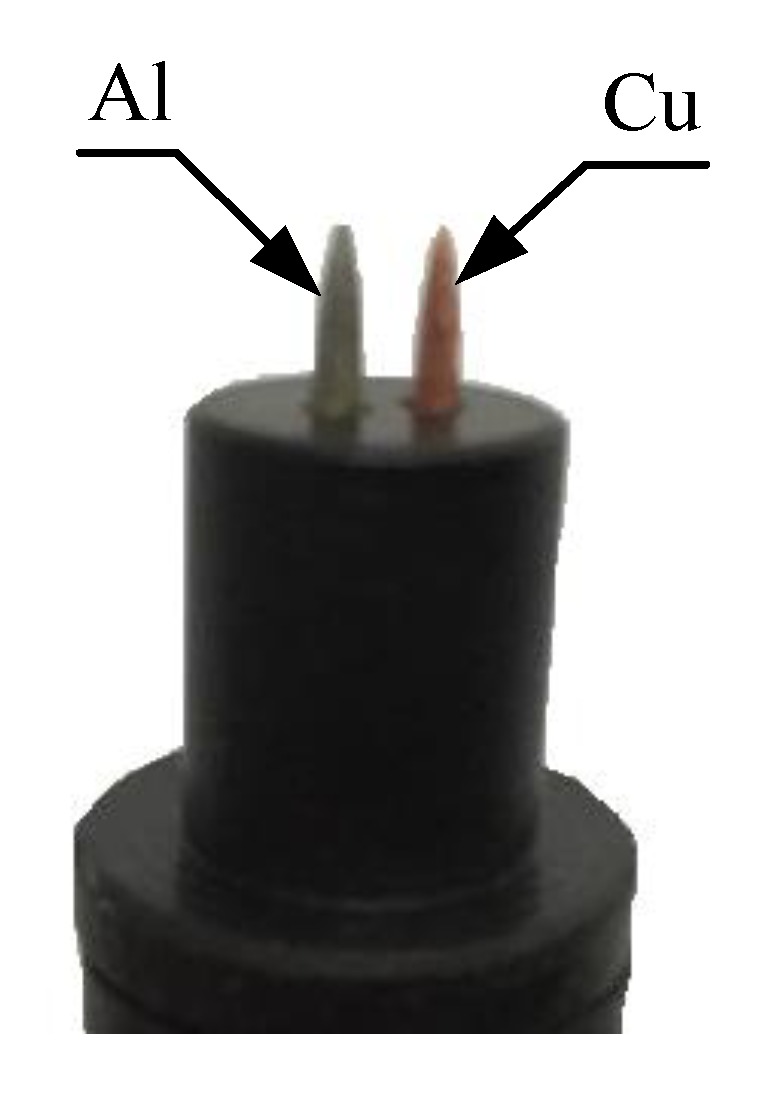
Structure of sensor head.

The electrodes used in this study act as sensors that detect electrolyte properties of the fruit and, consequently, the load resistance voltage is generated from the fruit battery. For this study, the electrodes are constructed properly using aluminum and copper metals because they are easily obtained and can be molded into the desired sensor head design as shown in Figure 4.

## 3. Characteristics of Fruit Battery with Charging Concept

### 3.1. Materials Preparation

The experiment was conducted using fresh oil palm fruits taken on the same day of each sets of experiment. In total, about 70 oil palm fruitlets were tested for different experimental purposes, such as investigation on fruitlets from different trees at different location, changing charging time and voltage, and changing release time. Specifically, for this study, about 30 fruitlets in total were tested for unripe, under ripe, and ripe categories of fruits. Over ripe fruitlets are not tested because of the evident number of loose fruits sockets found on the bunches. Furthermore, over ripe fruits can easily fall from the bunches while being handled at the mills. Thus, almost empty bunches are evident, and workers can simply reject the bunches at the screening process. Therefore, it is not necessary to develop load voltage characteristics of over ripe fruits for the sensor.

The fruitlets were selected using visual inspection based on the conventional grading standard set by the Malaysian Palm Oil Board (MPOB), which is widely used by Malaysia’s harvesters. There are a few methods to determine the fruit maturity such as fruitlet surface color, color of the mesocarp of the fruit, the number of loose fruits found on the ground before FFB is harvested, and the number of loose fruit sockets on the bunch. For the practicality of this research, the fruits were picked based on fruitlet surface color and the number of loose fruits found on the ground below the tree following the MPOB standard. Similarly, harvesters at the plantations can confirm the ripeness of FFB on the trees using these two visual methods simultaneously while workers at the screening area in the mills grade the maturity based on the same color inspection method and loose fruits sockets on the bunches.

Table 1 summarizes the characteristics of oil palm fruits based on the standard set by MPOB. Unripe fruits’ surface color was black to dark purple while under ripe fruits’ surface color was a dark brown to dark orange color. Both type of these fruits had no loose fruits detected on the ground. Meanwhile, ripe fruits could be distinguished with the color of red-orange and many loose fruits were seen on the ground. On the other hand, over ripe fruits were evident due to the significant number of loose fruits found on the ground at the plantations and visible empty sockets found on the bunch.

### 3.2. Experimental Setup

**Figure 5:**
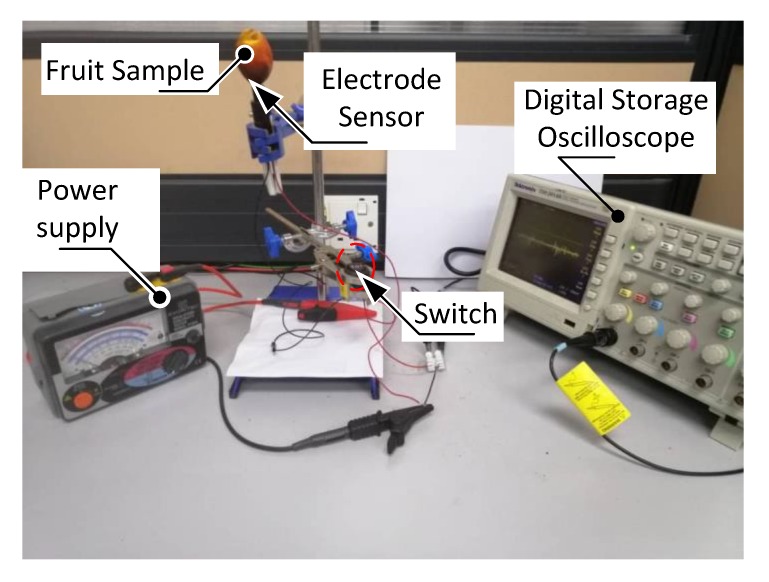
Photograph of experimental setup.

Figure 5 shows the photograph of the experimental setup. The fruit sample was thrusted onto the electrode sensors and it was connected to both the digital storage oscilloscope (DSO) and power supply used for charging of fruit battery. The DSO used in this experiment was the Tektronix TDS2014B that reads the voltage waveform signals (*y*-axis) as function of time (*x*-axis) and stored these signals as numerical values. As for the power supply used for charging of the fruit battery, an electrical insulation-continuity tester Model 3132A by Kyoritsu (Tokyo, Japan) was used. This equipment is typically used to test insulation performance of a cable by passing through electrical current at desired voltage. In this experiment, the tester was set at 250 V/100 MΩ, which represents the power supplied to the fruit battery. A switch labelled with number 1 and 2 was used to control which circuit was to be turned on during the experiment.

**Figure 6:**
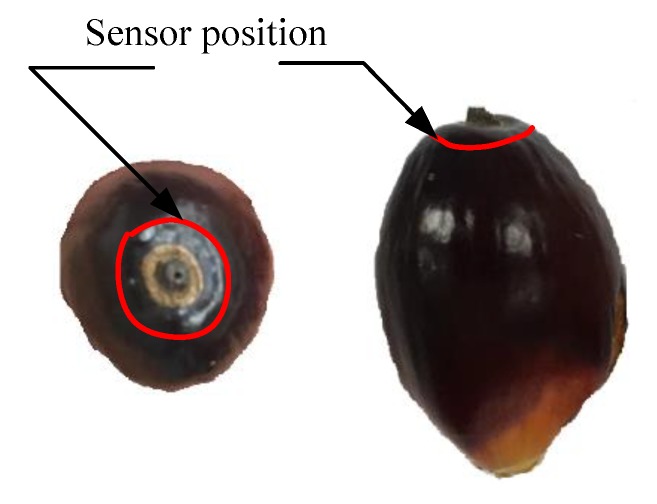
Electrode sensor position on the fruit surface.

The sensor position is illustrated in Figure 6 indicated with a circle line drawn on the top surface of the fruitlet. Each fruit as tested five times with each probe of the electrode sensor adjusted to another position anywhere on the circle line. A total of about five fruits were tested for each of the fruit maturity category, which are unripe, under ripe, and ripe. By switching on Switch 1, each fruit was subject to charging for 10 s before the fruit battery circuit was turned on by Switch 2 for another 30 s. The load resistance voltage readings were generated for the period of time when Switch 2 was turned on. As for the moisture content of the fruits, they were tested after each fruit load resistance voltage reading was recorded. An infrared moisture determination balance FD-610 by Kett Electric Laboratory (Tokyo, Japan) is used for weight analysis to measure the moisture content.

### 3.3. Performance Evaluation of Fruit Battery with Charging Concept

**Figure 7:**
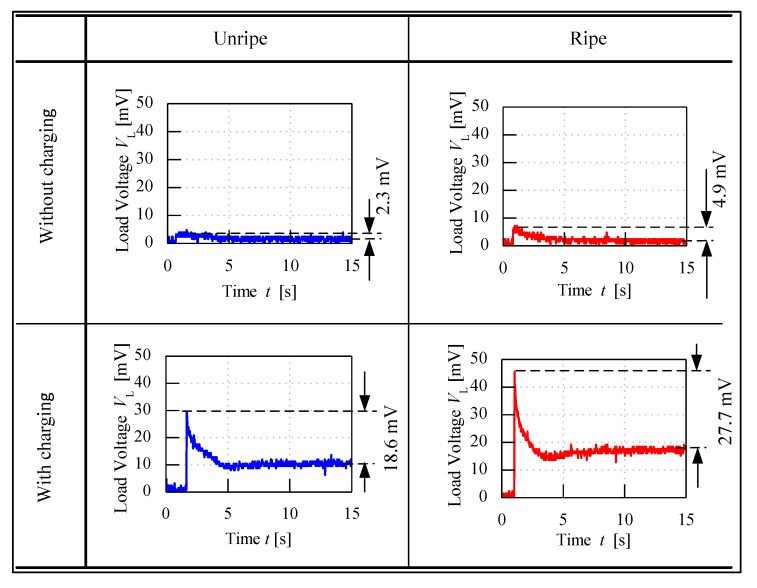
Load resistance voltage characteristics of fruit battery with and without charging process.

A control set of experiment using unripe and ripe fruits was done to show the significance behind charging process of the fruit. The electrode sensor was thrusted through the mesocarp part of the fruit and load voltage readings were produced. Based on Figure 7, the load resistance voltage characteristics of charged and without charging of unripe and ripe fruit can be better understood. For both conditions of charging and without charging process beforehand, when the fruit battery circuit was turned on for a period, the load voltage readings showed a maximum initial value when energy began to be discharged from the fruit battery and then reduced to the steady-state voltage as the energy release reached a stable discharge. The difference of load resistance voltage between the maximum peak and the steady-state condition, also known as effective load resistance voltage, ∆*V_L_* was a significant variable to distinguish between fruit maturity grade. 

From Figure 7, load voltage of both unripe and ripe fruits without charging process showed very similar values of ∆*V_L_*, which are 2.3 and 4.9 mV, respectively. The difference of ∆*V_L_* was very small between the fruits, hence they cannot be significantly distinguished. Meanwhile, when the fruit was charged beforehand, the load resistance voltage readings were amplified, and the maximum values were very different between unripe and ripe fruits, which resulted in large difference of ∆*V_L_* as well, of 18.6 and 27.7 mV, respectively. 

**Figure 8:**
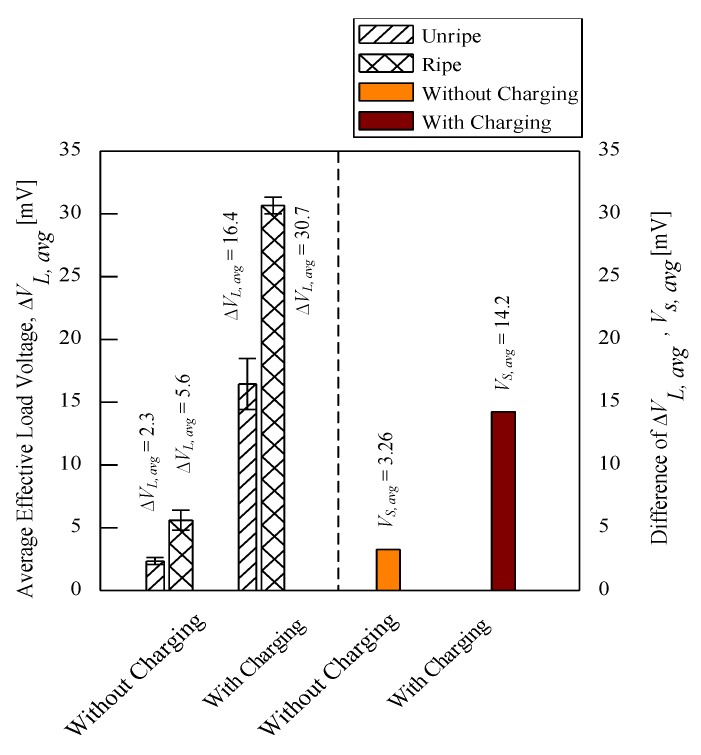
Performance comparison between charging and without charging process.

The significance of charging concept is further explained using another set of control experiments tested on five fruits for each unripe and ripe maturity grade. The electrode sensor was thrusted onto each fruit three times and the average load voltage values were calculated. Figure 8 illustrates the sensitivity of load resistance voltage readings for both unripe and ripe fruits. The average effective load voltage, ∆*V_L_*_,avg_, for both fruits without the charging process were very small compared to that of the fruits subjected to the charging process. The difference of average effective load voltage, ∆*V_L_*_,avg_, known as *V_s_*_,avg_, between the fruits that did not undergo charging process was only 3.26 mV compared to 14.2 mV for the fruits that were charged beforehand. In conclusion, the sensitivity of load voltage readings increased more than a factor of 4 after the charging process. In addition, the difference of effective load voltage readings between unripe and ripe fruits is evidently significant as seen by the error bars that did not overlap between both fruits that undergo charging process. Hence, this shows that the fruits maturity grade can be characterized with reference to their load voltage characteristics. 

### 3.4. Characteristics of Various Fruit Maturity Grade

**Figure 9:**
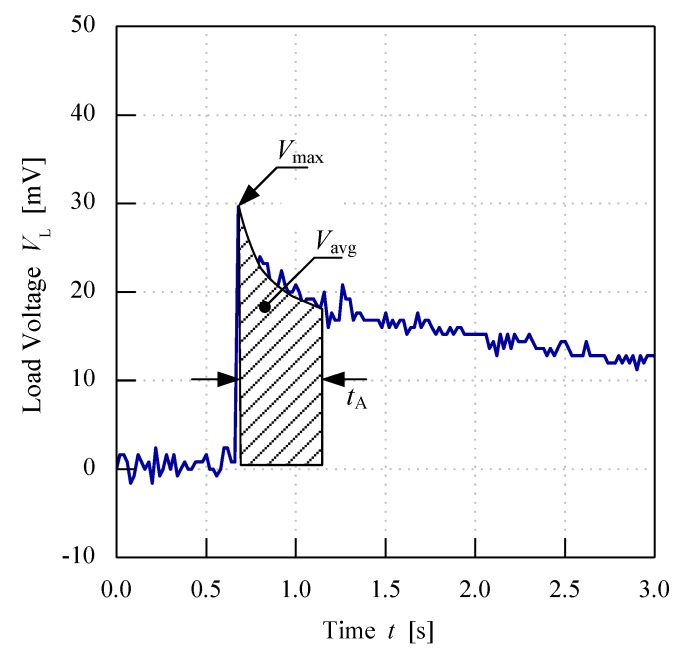
Methods to characterize load resistance voltage readings.

The load voltage readings were recorded by the oscilloscope and an example of the graph is shown in Figure 9. Due to the charging procedure introduced to the fruit, the load voltage readings for the fruit battery indicated an amplified voltage at the initial moment of energy release shown by the sharp peak of the graph. This peak or maximum value, *V*_max_, can be used as one of the indicators to classify each fruit maturity grade. However, *V*_max_ can be imprecisely identified from the graph due to unstable discharge of energy at the initial moment of energy release, thus resulting in fluctuations in voltage readings near the peak values. Therefore, another method can also be applied by taking the average load resistance voltage values near the maximum points, *V*_avg_. This is calculated from the area under the graph for points near to the peak values under 1 s of time interval as shown in Figure 9. Both values, *V*_max_ and *V*_avg_, are distinctive for each fruit maturity category; hence, they can be used to classify the fruits. 

From Table 2, a summary for the characteristics of the fruit maturity grade is presented. As discussed earlier, it is important to understand that this method depends on manual visual inspection based on FFB color as a preliminary screening in combination with the sensor that provides apparent indication of the fruit maturity grade. This table is a guide to develop the sensor system, which strongly depended on the voltage values. From the table, four categories of fruit maturity grade were characterized based on their *V*_avg_, *V*_max_, moisture content, *w* (%), and fruitlet surface color. The FFB appearance for each maturity grade based on MPOB standard was included in the table because the workers at the screening area follow the same standard during visual inspection. Unripe FFB are easily rejected through color inspection due to the apparent black/dark purple color of the fruits while over ripe FFB show significant numbers of loose sockets on the bunches. In addition, due to having similar appearance for under ripe and ripe fruits, this table helps to classify both type of fruits based on the load voltage characteristics.

Unripe fruits have lowest *V*_avg_ and *V*_max_ values of 13.0 and 19.9 mV, respectively when compared to under ripe and ripe 1 category, while the moisture content, *w*%, of the fruits showed a decreasing trend from 86.9 to 24.6%. As the fruit ripens, the oil content increases while the moisture content in the fruit decreases as they are converted into lipids where the ripe fruit’s flesh displays non-electrolyte properties [19]. This causes the load voltage values for certain ripe fruits to be lower due to sensor inability to properly measure them. This is observed from *V*_avg_ and *V*_max_ values for the ripe 2 grade; 17.4 and 27.2 mV, respectively, which are significantly lower than ripe 1 and under ripe, and quantitatively did not show any relationship or dependence with the ripe 1 category. It must be understood that load voltage is not detected for specific chemical content of oil palm fruits. Especially for ripe fruits that contain different types of composition, it is not possible to know the specific content of the fruits that is being measured by the sensor. Ripe 2 category of results is considered as ripe fruits because the FFB appearance is of ripe fruits (red–orange color and almost no loose fruits sockets on the bunch) and workers at the mills will identify these bunches as ripe fruits as well through visual inspection. 

Measuring moisture content can only be conducted indoor as a preliminary step to develop the classification. Using the calculated moisture content for each maturity grade, a threshold value for load voltage value of ripe fruits can be identified. In other words, a specific load voltage value was identified based on the minimum moisture content (*w*% < 44%) in order to classify ripe fruits from the other fruits. From the table, fruits with *V*_avg_ above 25 mV and *V*_max_ above 40 mV were considered as ripe. Consequently, two conditions can exist during usage of the sensor at the mills. When either one of the conditions is met, the workers can accept the tested bunch as ripe and it should be processed at the mill. Condition 1 occurs when the sensor detected *V*_avg_ above 25 mV or *V*_max_ above 40 mV when testing on the bunch. Meanwhile, condition 2 occurs when the appearance of the FFB is that of a ripe fruit as set by MPOB standards. For FFB that the worker failed to classify as ripe or under ripe, the sensor can then be used and condition 1 will apply.

### 3.5. Relationship of Fruit Moisture Content and Load Voltage

**Figure 10:**
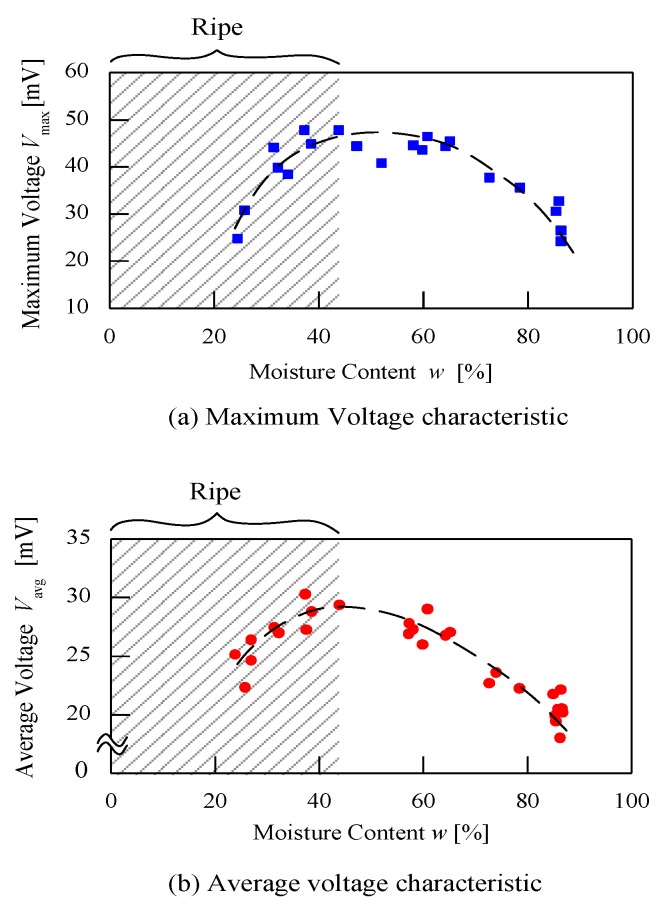
Relationship between moisture content and load resistance voltage. (**a**) Maximum voltage characteristic; (**b**) average voltage characteristic.

Based on moisture content, *w* (%), measured for each maturity grade, the characteristics of *V*_max_ and *V*_avg_ can be plotted as shown in Figure 10. In Figure 10a,b, a reference line with an arc shape can be seen clearly, which can evidently distinguish ripe fruits. As shown in Table 2, fruits with moisture content lower than 44% (*w* < 44%) were classified as ripe. Based on Figure 10, ripe fruits can be classified with *V*_max_ between 25 to 45 mV and *V*_avg_ between 20 to 30 mV. For unripe and under ripe fruit with moisture content more than 44% (*w* > 44%), the *V*_max_ and *V*_avg_ values were in different a range. From Figure 10b, *V*_avg_ characteristics for each maturity grade are evidently distinct especially for ripe fruits.

## 4. Conclusions

The current practice of the oil palm fruit grading system at milling factories utilizes human graders for visual inspection. This can lead to even more losses by sending bad-quality FFB due to inconsistent evaluation results and repeated mistakes. Therefore, a systematic system that can produce consistent results and an efficient screening process should be implemented at the mills. This paper presents an electrode sensor that can detect oil palm fruit maturity grade and send the signal indication to the user conducting the screening process at the mills, indicating whether to accept or reject the bunches. The research focused on application of the fruit battery principle to oil palm fruit and applying the concept of charging of battery to the fruit. The electrode sensor can detect amplified load voltage after the fruits are charged and the difference of effective load voltage, ∆*V_L_*_,avg_, between charged unripe and ripe fruits, *V_s_*_,avg_, is 14.2 mV compared to 3.26 mV for fruits that are not charged beforehand. This shows that the charging process resulted in significant load voltage readings that are more than 4 times sensitive than the results for uncharged fruits. Hence, a classification for the fruit maturity grade can be determined based on the load voltage values and the fruit moisture content. It is concluded that fruits with *V*_avg_ above 25 mV and *V*_max_ above 40 mV are considered to be ripe. In addition, the classification showed a very distinct character for the ripe fruit category whereby the fruits with moisture content, *w*, less than 44% with *V*_max_ between 25 to 45 mV and *V*_avg_ between 20 to 30 mV are classified as ripe fruits. For future work, an optimization study on resistance load, charging voltage, and charging period can be investigated to further define the characteristics of all oil palm maturity grade.

## Figures and Tables

**Table 1:** MPOB oil palm fruit maturity grading standard [20].

Fruit Maturity Grade	Unripe	Under Ripe	Ripe	Over Ripe
Fruitlet Surface Color	Black to dark purple	Dark brown to dark orange	Dark red to yellow–orange	Orange
Loose Fruits on Ground	0	0 to 5	>10	>50% of fruit
Loose Fruit Sockets on Bunch	0	<10	>10	>50% of fruit
Fruit Mesocarp Color	Yellow	Yellow–orange	Yellow–orange	Yellow–orange

**Table 2:** Characteristics of various fruit maturity grade.

Maturity Grade	Characteristics	Values			FFB Appearance
		*V*_avg_ (mV)	*V*_max_ (mV)	*w* (%)	
Unripe	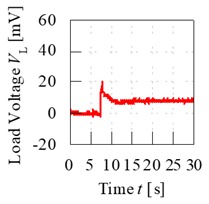	13.0	19.9	86.9	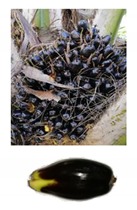
Under Ripe	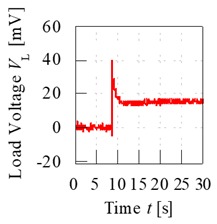	24.9	39.0	59.0	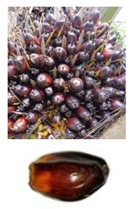
Ripe 1	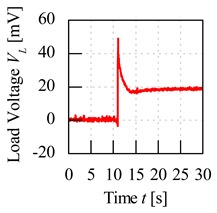	29.3	48.9	44.0	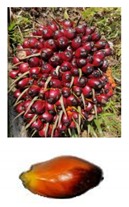
Ripe 2	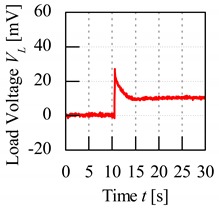	17.4	27.2	24.6	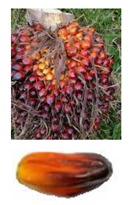

## References

[1] Nambiappan B. (2018). Malaysia: 100 Years of resilient palm oil economic performance. J. Oil Palm Res..

[2] Kushairi A., Ong-Abdullah M., Nambiappan B., Hishamuddin E., Izuddin Z., Ghazali R., Subramaniam V., Sundram S., Parveez G.K.A. (2019). Oil Palm Economic Performance in Malaysia and R&D Progress in 2018. J. Oil Palm Res..

[3] Chang L.C., Abdul R.A.S., Zainon B. (2003). An economic perspective of oil extraction rate in the oil palm industry of Malaysia. Oil Palm Ind. Econ. J..

[4] Zulkefli F., Othman N., Syahlan S., Zaini M.R., Bakar M.A. (2017). Fresh Fruit Bunch Quality and Oil Losses in Milling Processes as Factors that Affect the Extraction Rate of Palm Oil. Int. J. Agric. For. Plant..

[5] (2017). FGV Targets 22% Oil Extraction Rate by 2020. https://www.thestar.com.my/business/business-news/2017/03/17/fgv-targets-22pc-oil-extraction-rate-by-2020/.

[6] Abdullah M.Z., Guan L.C., Mohd Azemi B.M.N. (2001). Stepwise Discriminant Analysis for Colour Grading of Oil Palm Using Machine Vision System. Food Bioprod. Process..

[7] Fadilah N., Saleh H.M., Halim Z.A., Ibrahim H., Salim S., Ali S. (2012). Intelligent Color Vision System for Ripeness Classification of Oil Palm Fresh Fruit Bunch. Sensors.

[8] May Z., Amaran M.H. (2010). Automated Ripeness Assessment of Oil Palm Fruit Using RGB and Fuzzy Logic Technique. Inf. Technol..

[9] Harun N.H., Misron N., Sidek R.M., Aris I., Ahmad D., Wakiwaka H., Tashiro K. (2013). Investigations on a novel inductive concept frequency technique for the grading of oil palm fresh fruit bunches. Sensors.

[10] Ismail W.I.W., Razali M.H., Ramli A.R., Sulaiman M.N., Harun M.H.B. (2009). Development of imaging application for oil palm maturity prediction. Eng. E-Trans..

[11] Balasundram S.K., Robert P.C., Mulla D.J. (2006). Relationship between oil content and fruit surface color in oil palm (Elaeis guineensis Jacq.). J. Plant Sci..

[12] Jamil N., Mohamed A., Abdullah S. Automated Grading of Palm Oil Fresh Fruit Bunches (FFB) Using Neuro-Fuzzy Technique. Proceedings of the 2009 IEEE International Conference of Soft Computing and Pattern Recognition.

[13] May Z., Amaran M.H. (2011). Automated oil palm fruit grading system using artificial intelligence. Int. J. Eng. Sci..

[14] Alfatni M.S.M., Shariff A.R.M., Shafri H.Z.M., Saaed O.M.B., Eshanta O.M., Abuzaed M. Automated Oil Palm Fruit Bunch Grading System Using Density of Color (RGB). Proceedings of the 7th Saudi Engineering Conference.

[15] Alfatni M.S.M., Shariff A.R.M., Shafri H.Z.M., Saaed O.M.B., Eshanta O.M. (2008). Oil palm fruit bunch grading system using red, green and blue digital numbers. J. Appl. Sci..

[16] Jaffar A., Jaafar R., Jamil N., Low C.Y., Abdullah B. (2009). Photogrammetric grading of oil palm fresh fruit bunches. Int. J. Mech. Mechatron. Eng..

[17] Junkwon P., Takigawa T., Okamoto H., Hasegawa H., Koike M., Sakai K., Siruntawineti J., Chaeychomsri W., Sanevas N., Tittinuchanon P. (2009). Hyperspectral imaging for nondestructive determination of internal qualities for oil palm (Elaeis guineensis Jacq. Var. tenera). Agric. Inf. Res..

[18] Sharifudin M.S., Cardenas-Blanco A., Gao Amin M.H., Soon N.G., Laurance D.H. (2010). Monitoring development and ripeness of oil palm fruit (Elaeis guineensis) by MRI and bulk NMR. Int. J. Agric. Biolog..

[19] Minakata K., Tashiro K., Wakiwaka H., Kobayashi K., Misron N., Aliteh N.A., Nagata H. Proposal of Fruit Battery Method for Estimating Oil Palm Ripeness. Proceedings of the 2018 12th International Conference on Sensing Technology (ICST).

[20] MPOB, Ministry of Plantation Industries and Commodities (2015). Grading Procedures. Oil Palm Fruit Grading Manual.

